# Development of an Initial Conceptual Model of Multiple Myeloma to Support Clinical and Health Economics Decision Making

**DOI:** 10.1177/2381468318814253

**Published:** 2019-01-17

**Authors:** Sebastian Gonzalez-McQuire, Meletios-Athanassios Dimopoulos, Katja Weisel, Walter Bouwmeester, Roman Hájek, Marco Campioni, Craig Bennison, Weiwei Xu, Krystallia Pantiri, Marja Hensen, Evangelos Terpos, Stefan Knop

**Affiliations:** Amgen (Europe) GmbH, Rotkreuz Switzerland; National and Kapodistrian University of Athens School of Medicine, Athens, Greece; University Hospital of Tübingen, Tübingen, Germany; Pharmerit International, Rotterdam, Netherlands; Department of Hematooncology, University Hospital Ostrava, Ostrava, Czech Republic; Faculty of Medicine, University of Ostrava, Ostrava, Czech Republic; Amgen (Europe) GmbH, Rotkreuz Switzerland; Pharmerit Ltd, York, UK; Pharmerit International, Rotterdam, Netherlands; Pharmerit International, Rotterdam, Netherlands; Pharmerit International, Rotterdam, Netherlands; National and Kapodistrian University of Athens School of Medicine, Athens, Greece; Würzburg University Medical Center, Würzburg, Germany

**Keywords:** conceptual model, Delphi panel, economic modeling, multiple myeloma, systematic literature review

## Abstract

**Background.** We aimed to develop and validate a conceptual model of multiple myeloma (MM) that characterizes the attributes affecting disease progression and patient outcomes, and the relationships between them. **Methods.** Systematic and targeted literature reviews identified disease- and patient-specific attributes of MM that affect disease progression and outcomes. These attributes were validated by a Delphi panel of four international MM experts, and a physician-validated model was constructed. Real-world clinical data from the Czech Registry of Monoclonal Gammopathies (RMG) was used to confirm the relationships between attributes using pairwise correlations and multiple Cox regression analysis. **Results.** The Delphi panel reached consensus that most cytogenetic abnormalities influenced disease activity, which results in symptoms and complications and affects overall survival (OS). Comorbidities and complications also affect OS. The entire panel agreed that quality of life was influenced by comorbidities, age, complications, and symptoms. Consensus was not reached in some cases, in particular, the influence of del(17p) on complications. The relationships between attributes were confirmed using pairwise analysis of real-world data from the Czech RMG; most of the correlations identified were statistically significant and the strength of the correlations changed with successive relapses. Czech RMG data were also used to confirm significant predictors of OS included in the model, such as age, Eastern Cooperative Oncology Group performance status, and extramedullary disease. **Conclusions.** This validated conceptual model can be used for economic modeling and clinical decision making. It could also inform the development of disease-based models to explore the impact of disease progression and treatment on outcomes in patients with MM.

Multiple myeloma (MM) is a common hematological malignancy, accounting for up to 10% of hematological cancers and 1% of all cancers.^1,2^ It is characterized by aberrant clonal expansion of plasma cells within the bone marrow and the secretion of large amounts of immunoglobulin, known as M protein.^3^ Increased use of new agents for the treatment of patients with MM has improved survival. For example, in Germany, age-adjusted 5-year survival for those with newly diagnosed MM increased from 47.3% between 2004 and 2008 to 53.8% between 2008 and 2012.^4^ In the United Kingdom, 1-, 5-, and 10-year age-adjusted survival of patients with MM diagnosed between 2010 and 2011 was 76.6%, 47%, and 32.5%, respectively.^5^ Inevitably, all patients with MM will experience relapses and many will undergo multiple lines of treatment.^6^ Because of the heterogeneity of the disease, treatment responses vary and are often affected by treatment-related toxicity or complications arising from the natural progression of the disease.^1,7^ Indeed, a study of the clinical course of patients with MM reported that the duration of response decreased consistently with each line of therapy, and that 84% of patients died within 5 years of first relapse.^8^ MM may also become refractory to treatment.^9^

Treatment has evolved considerably from the previous standards of care using alkylating agents plus steroids, and now includes novel targeted agents and high-dose chemotherapy and stem cell transplantation (SCT) for patients aged ≤70 years.^10^ Relapse rates are high and most patients will receive a new anti-MM agent, several of which are now approved in Europe, including the proteasome inhibitors bortezomib,^11^ carfilzomib,^12^ and ixazomib^13^; immunomodulatory drugs such as thalidomide,^14^ lenalidomide,^15^ and pomalidomide^16^; the monoclonal antibodies daratumumab^17^ and elotuzumab^18^; and the histone deacetylase inhibitor panobinostat.^19^

Choice of therapy is influenced by, among other things, approval status, availability of, and reimbursement guidelines for MM drugs, and also patient preference and suitability for treatment. Disease- and patient-related factors, and response to previous therapies, are particularly important for those with relapsed or refractory disease because there is no generally accepted standard of care for these patients.^10^ Heterogeneity among patients with MM means that treatment responses may vary; however, there is a lack of information on the specific patient populations that will respond to certain treatment regimens. Furthermore, patients with MM often have comorbidities such as renal impairment or peripheral neuropathy, which should be considered when deciding on treatment.^10^ A better understanding of how these individual factors and their interrelationships influence the progression of MM and patient outcomes would aid the assessment of new interventions. These concepts can be assimilated into a model that may help improve our knowledge of different aspects of the MM disease process from clinical and economic viewpoints.

A conceptual model is a simplified representation of reality that informs medical decisions and perceptions of prognosis, which can provide the basis for health economic modeling.^20^ The International Society for Pharmacoeconomics and Outcomes Research (ISPOR) and the Society for Medical Decision Making (SMDM) Task Force recommend that a conceptual model is developed before constructing an economic model, to ensure that all key components and endpoints have been identified.^20–23^ Evidence on clinical and economic outcomes is structured to help decision makers evaluate health care interventions. Therefore, a conceptual model of MM could be used to predict long-term outcomes for those with the disease. Conceptual models in other disease areas (e.g., chronic obstructive pulmonary disease) have been reported,^23^ and Baz et al. developed a conceptual model looking at how MM and its treatment affect health-related quality of life.^21^ However, there is no comprehensive conceptual model that could bridge the gap between the factors involved in the disease process in MM, its progression, and subsequent effects on patient outcomes.

Our objective was to develop and validate a conceptual model of MM that can provide clinicians with a comprehensive framework of patient characteristics, leading to a better understanding of the attributes that influence disease progression and, ultimately, patient outcomes. We also aimed to define the interrelationships and potentially causal relationships among attributes. This conceptual model may improve understanding of the attributes that influence disease progression and consequently affect health economics decision making and patient management. For example, resource allocation may be determined based on this model, and the model may also be used as a basis for the development of economic models.

Disease- and patient-specific attributes that affect disease progression and outcomes were identified from published literature and were validated, together with their interrelationships, via a Delphi panel of experts in hemato-oncology. The physician-validated model was explored further using real-world data obtained from the Czech Registry of Monoclonal Gammopathies (RMG) to ensure that only relevant attributes were included. The Czech RMG is one of the largest hematological registries in Europe; it collects data from patients with hematological malignancies in the Czech Republic and Slovakia.^24^ Registry data provide valuable insights into treatment outcomes for patients in clinical practice, capturing a broader range of patients than in clinical trials. This model has the potential to be expanded to examine how the relationships between attributes change over time and with therapeutic intervention.

## Methods

### Literature Review

The first step of the modeling process involved conceptualization of the problem and the model,^25^ addressed through literature reviews and a Delphi panel, respectively. Systematic literature reviews identified studies relating to the disease burden of MM, economic models of MM, and clinical trials of MM treatments. The objective of the literature review, which took place over a 3-month period, was to identify all of the factors potentially related to the disease process in MM. These included patient characteristics, genetic factors, disease characteristics, and complications, together with various disease-related and patient-related outcome measures. A key aspect of this process was to capture the relationships between the different factors, because this information would be used to develop the conceptual model of MM. Search terms for the literature databases were therefore selected to focus on conceptual or disease models, and associations, correlations, or relationships between factors in MM.

Searches included the Embase and Medline (including PubMed) databases (2004–2014), annual meeting proceedings from the American Society of Clinical Oncology (ASCO) and American Society of Hematology (ASH) (2012–2014), health technology assessment (HTA) reports (2004–2014), and treatment guidelines for patients with MM (2009–2014). Databases searched and search terms used are presented in Online Appendix 1 (Tables S1–S6). The results of these searches formed the basis for model development, in line with guidance from the National Institute for Health and Care Excellence.^25^

Following removal of duplicates, titles and abstracts were screened and final inclusion of articles was based on review of the full text. The criteria for inclusion are summarized in Online Appendix 1 (Table S7). A data-extraction Microsoft Excel file was developed to organize the publications, and extracted data are listed in Online Appendix 1 (Table S8). The focus of this research was on non–treatment-specific attributes; therefore, adverse events were not included. Publications of guidelines and HTA reports that were not written in English would have been permitted if an English language version was available, but this was not necessary because none was identified.

### Development of the Conceptual Model

Components of the conceptual model were defined as follows.

- *Attribute:* A metric considered to be a characteristic or inherent part of the MM disease process. Attributes could be explanatory or dependent. A change in an explanatory attribute was considered to have a direct effect on a dependent attribute and its value (e.g., increases in age [explanatory attribute] were associated with an increased risk of death [dependent attribute]).
- *Attribute category:* A group of related attributes representing a particular patient characteristic or disease process.
- *Outcome:* A patient-related outcome that differs from a disease outcome, such as quality of life (QoL), that is directly or indirectly affected by other attributes.
- *Disease progression:* A disease outcome representing worsening disease that is directly or indirectly affected by other attributes.
- *Interrelationship:* Individual attributes or attribute categories that influence each other, directly or indirectly.
- *Causal relationship:* Individual attributes or attribute categories that have a direct effect on another attribute.

Key attributes used to measure and to define MM, and information on how these attributes can influence, or be influenced by, disease progression and patient outcomes in MM, were identified from the literature reviews. The attributes and outcomes were grouped according to their interrelationships, and the groups were linked according to the relationships that each might have with the others. The groups were subsequently organized into broad categories, such as disease characteristics, patient characteristics, and key outcomes.

### Delphi Panel

The initial draft of the conceptual model was assessed and validated in a stepwise manner using the Delphi method. This is a widely used and accepted group communication process that aims to achieve a convergence of opinion on a specific real-world issue.^26^ The Delphi panel comprised four practicing hematologists recognized as international experts in MM. The aims of the Delphi panel were primarily to identify and to qualify the most relevant disease attributes identified from the literature reviews that affect disease progression and patient outcomes, and subsequently to explore the potentially causal relationships between the attributes. Treatment effects were not explored.

One-to-one interviews were conducted with each Delphi panel member. Panel members prepared for the interview by reviewing the interview guide, which was also used during the interview (Online Appendix 2). The conceptual model was then revised to reflect the opinions of the experts and reviewed by the panel via a written assignment. Another round of interviews was conducted, during which consensus was sought for each attribute and association that had been incorporated into the model. Through this process the physician-validated conceptual model was constructed.

### Quantifying the Conceptual Model Using Real-World Data

A separate validation step was performed after completion of the literature review and Delphi panel process, using registry data not used in the development of the conceptual model. The attributes, outcomes, and relationships confirmed by the physicians were quantified using real-world data obtained from the RMG.^24^ If available, data on attributes and outcomes identified by the Delphi panel were obtained from Czech adults (≥18 years old) enrolled in the RMG who had been diagnosed with symptomatic MM between May 2007 and April 2016.^27^ If attribute data were not available, proxies were used where possible (e.g., the presence of two or more osteolytic lesions and a bone-related extramedullary mass were used as proxies for pain). Pairwise analyses were performed on the identified attributes at diagnosis and by treatment line to identify correlations between attributes. Pearson’s *R* correlation coefficients were calculated and statistical significance was set at *P* < 0.05.

An analysis of predictors of overall survival (OS) from the initiation of first-line treatment was performed to confirm the physician-validated attributes in the conceptual model for which data were also available in the RMG and to identify potential new variables that predicted OS.^28^ Attributes considered predictive of OS were fitted to a multiple Cox regression model and backward selection was performed using Akaike’s information criterion.^28^ Proxies were not used for unavailable attributes in the Cox model, and assessment of confounding was limited to variables associated with OS.

## Results

### Literature Reviews

**Figure 1:**
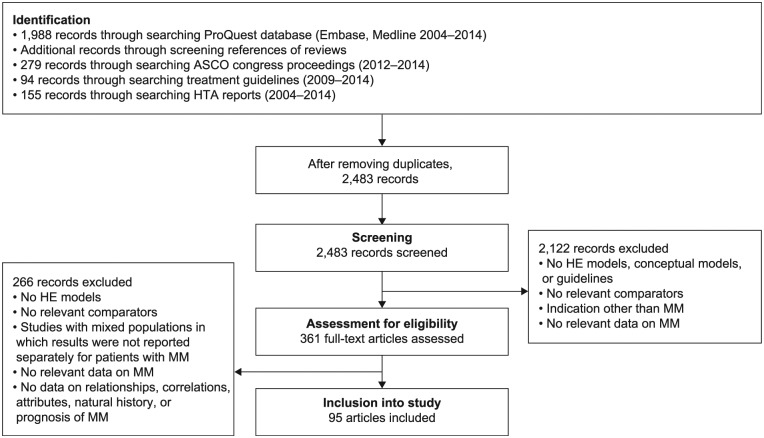
PRISMA flow chart of the systematic literature searches. ASCO, American Society of Clinical Oncology; HE, health economics; HTA, health technology assessment; MM, multiple myeloma; PRISMA, Preferred Reporting Items for Systematic Reviews and Meta-Analyses.

The ProQuest search of PubMed and Embase databases and ASH congress proceedings identified 1,988 relevant papers; another 279 abstracts were identified from the search of ASCO congress proceedings. In addition, 155 HTA reports and 94 guidelines were identified. Following removal of duplicates, 2,483 records were screened and 2,122 discarded, leaving 361 to be screened for eligibility by assessment of the full text (if available). In total, 131 records (89 articles [36 papers and 53 abstracts], 30 HTA reports, and 12 guidelines) met the inclusion criteria (Figure 1). The records included case–control studies, chart reviews, cohort studies, retrospective studies, database analyses, clinical trials, patient surveys, reviews, and original research from the European Union, the United States, Canada, Asia, Egypt, and Brazil. Most studies included patients with newly diagnosed MM.

**Figure 2:**
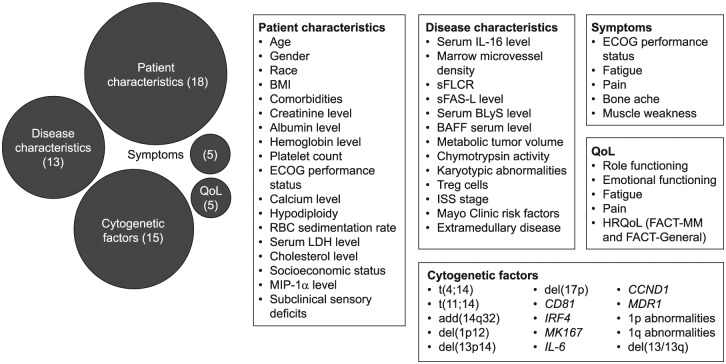
Variables in each category that have a significant relationship with another attribute. BAFF, B-cell activating factor; BLyS, B-lymphocyte stimulator; BMI, body mass index; *CCND1*, gene encoding cyclin D1; *CD81*, gene encoding cluster of differentiation 81; ECOG, Eastern Cooperative Oncology Group; ESR, erythrocyte sedimentation rate; FACT-MM, Functional Assessment of Cancer Therapy–Multiple Myeloma; HRQoL, health-related quality of life; IL, interleukin; *IRF4*, gene encoding interferon regulatory factor 4; ISS, International Staging System; LDH, lactate dehydrogenase; *MDR1*, multidrug resistance gene 1; MIP-1α, macrophage inflammatory protein 1α; *MKI67*, gene encoding marker of proliferation Ki-67; MM, multiple myeloma; QoL, quality of life; RBC, red blood cell; sFAS-L, soluble Fas ligand; sFLCR, serum free light-chain ratio. Numbers in brackets are the number of attributes in each group. In total, 56 MM attributes were identified as significant, that is, those that have a significant relationship with another attribute; these were grouped into five categories: disease characteristics,^44–54^ cytogenetic factors,^55–59^ patient characteristics,^45–48,51,53,55,60–81^ QoL,^69,75,82,83^ and symptoms.^46,69,71,82–87^

Of the 97 MM attributes that were identified from the literature reviews, 56 significant attributes were selected, that is, those that had a significant relationship with another attribute. These were grouped into five attribute categories: disease characteristics, cytogenetics, patient characteristics, QoL, and symptoms. Figure 2 shows the significant attributes identified in each category.

**Figure 3:**
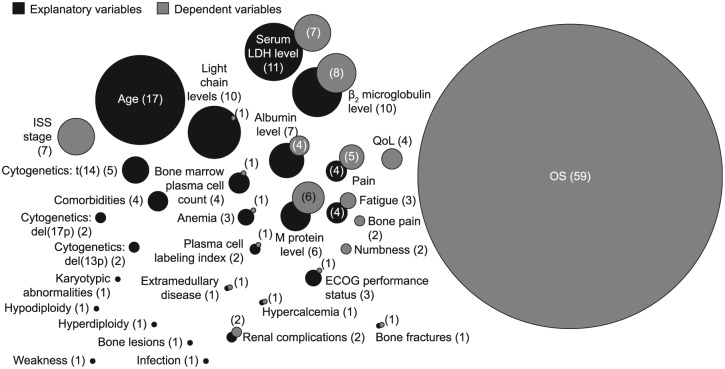
Explanatory and dependent variables identified from literature reviews. ECOG, Eastern Cooperative Oncology Group; ISS, International Staging System; LDH, lactate dehydrogenase; OS, overall survival; QoL, quality of life. From the attributes identified, 26 were deemed explanatory variables and 20 were dependent variables. Numbers in brackets are the number of times that the model attribute was featured in the findings of the literature search. Overlap of circles means that an attribute was found to be both a dependent and explanatory attribute. Explanatory variables: age,^46–48,60,61,63–71,74,77,80^ serum LDH level,^46,51,62,64,65,73,76,79–81,88^ light chains level,^44,46–54^ β_2_ microglobulin level,^51,62,64,70,71,79–81,89,90^ albumin level,^53,64–66,71,79,80^ M protein level,^59,70,91–93^ t(14),^55–59^ pain,^69,83,85,87^ bone marrow plasma cell count,^64,71,80,94^ comorbidities,^45,63,76,78^ fatigue,^69,82,83,87^ ECOG performance status,^46,71,75^ anemia,^51,65,72^ del(13p),^55,57^ del(17p),^55,57^ plasma cell labeling index,^58,95^ renal complications,^72,76^ hypodiploidy,^55^ hyperdiploidy,^96^ karyotypic abnormalities,^59^ extramedullary disease,^45^ hypercalcemia,^65^ bone lesions,^84^ weakness,^83^ bone fractures,^69^ and infection.^69^ Dependent variables: OS,^45–47,49–51,54–61,63–65,67,68,71–74,76,78–81,87–90,96–122^ β_2_ microglobulin,^55,73,106,109,123–126^ serum LDH level,^62,88,112,117,120,123,126^ ISS stage (albumin, β_2_ microglobulin),^55,70,109,117,123,126,127^ M protein level,^59,90,92,124,128^ pain,^75,86,129–131^ albumin level,^55,109,117,123^ QoL,^69,75,82,83^ fatigue,^85,129,130^ numbness,^130,131^ renal complications,^53,65^ bone pain,^129,130^ bone fractures,^132^ hypercalcemia,^133^ extramedullary disease,^102^ ECOG performance status,^75^ anemia,^93^ light chains level,^118^ plasma cell labeling index,^95^ and bone marrow plasma cell count.^124^

Following the first Delphi panel round, categorization identified 26 explanatory variables and 20 dependent variables (Figure 3). The most commonly reported explanatory variables included age, International Staging System (ISS) stage, and levels of serum lactate dehydrogenase (LDH), immunoglobulin light chains, β_2_ microglobulin, albumin, and serum and urine M protein. The most commonly reported dependent variable was OS, followed by QoL. None of the studies reviewed presented a comprehensive set of determinants of disease progression and outcomes, and no conceptual models linking factors involved in the disease process, its progression, and/or patient outcomes were identified.

### Development of the Initial Conceptual Model Using Physician-Driven Attributes

The Delphi panel considered the patient and disease characteristics that might influence the MM disease process identified through the literature reviews. There was consensus that the categories defined in the model, and the attributes assigned to each group, were correct. However, agreement was not reached on the interrelationships between some attributes within groups, or attribute categories as a whole. This reflects the heterogeneous nature of the disease and limited evidence on the associations between such attributes and their effect on the disease process.

All 97 attributes identified were considered by the panel in the first round of interviews, to ensure that potentially important variables that may have been of interest to the experts were not excluded prospectively. The first-round interviews identified OS and QoL as important outcomes; physical activity, psychological fitness, and comorbidities were important aspects of QoL. Although complete agreement was not reached on which parameters belonged in the patient/disease characteristics group, the panel members agreed that patient and disease characteristics should not be separated, and that cytogenetic factors could be grouped together. Most panel members considered important patient/disease characteristics to be age, Eastern Cooperative Oncology Group (ECOG) performance status, ISS stage, extramedullary disease status, serum free light-chain ratio, and levels of serum calcium, serum LDH, immunoglobulin subtypes (G, A, D, kappa/lambda light chain), and bone marrow plasma cells. Hypoploidy, karyotype abnormalities, t(4;14), t(14;16), del(17p), 1p, and 1q abnormalities were considered by most panel members to be important cytogenetic factors. The experts agreed that symptoms cannot be separated from complications, because most of the symptoms of MM are caused by complications. The experts also agreed that anemia, breathlessness and paleness, bone lesions and/or fractures, bleeding, infections, kidney damage, neuropathy, and pain are important symptoms or complications. Disease progression should be measured with a set of indicators, including CRAB criteria (hypercalcemia, renal insufficiency, anemia, and lytic bone lesions or osteoporosis), M protein level, serum light chain level, and extramedullary disease (mass) detected by imaging. Although M protein is a signal of tumor load in most patients, it is not a good measure for those with non-secretory disease.

Consensus was not reached for many of the associations and required further consideration; this was achieved through a written assignment. Table 1 shows the frequency and direction of associations agreed by at least 50% of the Delphi panel. It was agreed that “tumor activity/growth” could be used as a composite measure of disease progression. Consensus was reached on positive associations between extramedullary disease and disease progression and tumor activity/growth, and between serum LDH and tumor activity/growth, and on negative associations between kidney damage and OS, and for pain and ambulation and mobility. Of the 50 associations between attributes identified, agreement was reached by three of the four experts in 38% of cases (19 associations) and by two of the experts in 52% of cases (26 associations).

**Table 1:** Associations Between Attributes Agreed by at Least 50% of the Delphi Panel (*N* = 4)

Association	Frequency of Agreement, *n*
Positive associations	
Age & ECOG Performance Status	3
sFLCR & ISS Stage	2
Anemia & Infection	2
Bone Lesions & Bone Pain/Fracture	3
Infection & Kidney Damage	2
Neuropathy & Pain	2
Age & Anemia	2
Age & Infection	2
ECOG Performance Status & Pain	2
Serum Calcium & Bone Lesion/Fracture	3
sFLCR & Kidney Damage	2
ISS Stage & Kidney Damage	2
ISS Stage & Disease Progression	2
Calcium & Disease Progression	2
Serum LDH & Disease Progression	3
Extramedullary Disease & Disease Progression	4
Hypodiploidy & Disease Progression	3
t(4;14) & Disease Progression	3
del(17p) & Disease Progression	3
Independence & Infection	2
Calcium & Tumor Activity/Growth	2
Serum LDH & Tumor Activity/Growth	4
Extramedullary Disease & Tumor Activity/Growth	4
Karyotypic Abnormalities & Tumor Activity/Growth	2
Tumor Activity/Growth on All Symptoms/Complications	≥2^a^
Negative associations	
Anemia & OS	2
Anemia & QoL	≥2^a^
Bone Lesion & QoL	2
Bleeding & Work Life	2
Infection & OS	3
Kidney Damage & OS	4
Neuropathy & QoL	2
Pain & Ambulation and Mobility	4
Pain & Family and Family Life	2
Ambulation/Mobility & Fracture	3
Leisure/Hobbies & Infection	2
Usual Activities & Pain	2
Sex/Intimacy & Pain	2
Age & OS	3
ECOG Performance Status & OS	3
Serum LDH & OS	3
ISS Stage & OS	2
Extramedullary Disease & OS	3
Hypodiploidy & OS	3
Karyotypic Abnormalities & OS	2
t(4;14) & OS	3
t(14;16) & OS	3
del(17p) & OS	3
Tumor Activity/Growth on All Key Outcomes (OS & QoL)	≥3^a^
Tumor Activity/Growth on Disease Pathway	3

ECOG, Eastern Cooperative Oncology Group; ISS, International Staging System; LDH, lactate dehydrogenase; OS, overall survival; QoL, quality of life; sFLCR, serum free light-chain ratio.

a In some cases, agreement was reached on the association between attributes, but the reasoning behind the agreement differed between physicians.

**Figure 4:**
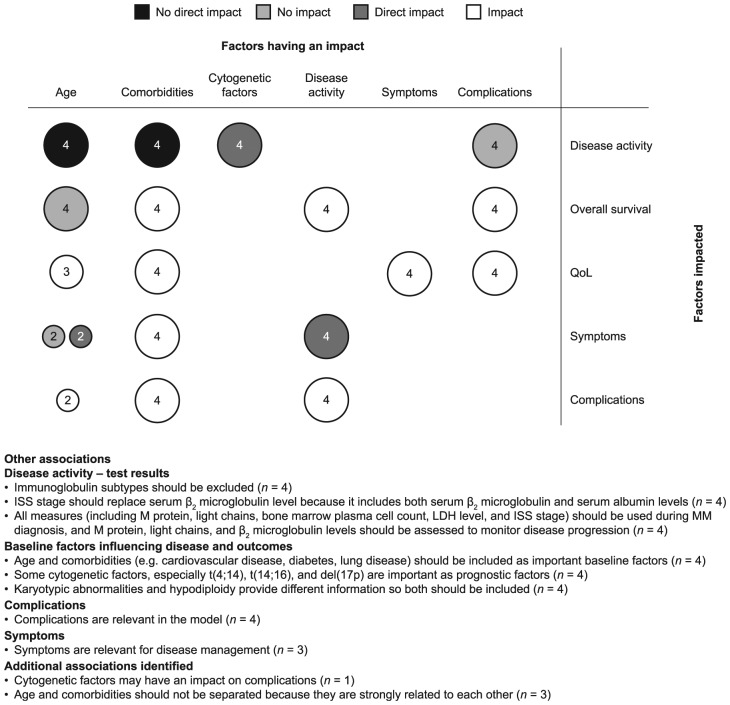
Associations between attributes agreed by the Delphi panel. ISS, International Staging System; LDH, lactate dehydrogenase; MM, multiple myeloma; QoL, quality of life. The *x*-axis shows factors that were agreed to either affect or not affect the factors on the *y*-axis. The size of the circles and the numbers within show the strength of the association in terms of how many Delphi panel members agreed. For example, age has no direct impact on disease activity (*n* = 4).

In the second round of interviews, all Delphi panel members agreed with the general structure of the conceptual model and the key attributes included. Patient characteristics were divided into cytogenetic factors, age, and renal comorbidities/ECOG performance status. Other areas of consensus/agreement reached by the panel during this step are summarized in Figure 4.

**Figure 5:**
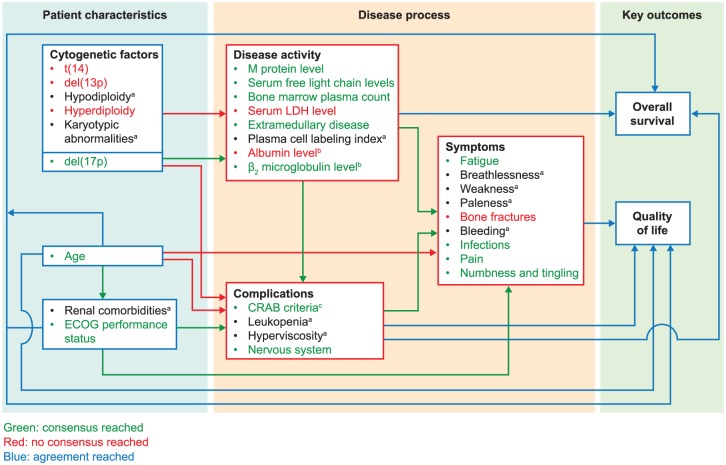
Map of associations between attributes that impact on disease progression and patient outcomes: results from the literature review and Delphi panel validation. CRAB, hypercalcemia, renal insufficiency, anemia, and lytic bone lesions or osteoporosis; ECOG, Eastern Cooperative Oncology Group; ISS, International Staging System; LDH, lactate dehydrogenase; MM, multiple myeloma. ^a^Insufficient data available. ^b^Estimated from ISS stage at diagnosis. ^c^CRAB criteria. Consensus was defined as agreement among all four panel members. Agreement was defined as 50% or more of panel members holding the same opinion (considered sufficient for this exercise because MM is a heterogeneous disease and it was important not to exclude relevant attributes at this early stage of model development). In addition, if only two of the Delphi panel members had the same opinion, the other two panel members were required to hold different opinions from each other for “agreement” to be reached. All included associations were agreed by at least 50% of the panel.

Consensus was not reached on the direct effect of cytogenetic factors on the disease process; in particular, t(4; 14) and del(17p) were considered to be important prognostic indicators but there was no consensus on their impact on disease activity. Cytogenetic factors may also influence complications, but consensus was not reached on this, and the suggestion was based on del(17p) only. Thus, del(17p) was added as a separate sub-box and linked to complications and disease activity. Age and comorbidities are heavily interlinked but have been separated in the model because the panelists did not agree about the relationship between age and complications and symptoms: some experts suggested a direct relationship, whereas others suggested an indirect relationship via comorbidities. In the physician-validated model, no consensus was reached on the relationship between age and symptoms and complications; consensus was reached on the impact of ECOG performance status and renal comorbidities on complications and symptoms (Figure 5).

Age was important in the model, but there was disagreement about its interrelationships with other attributes. Consensus was reached that age has an indirect effect on other attributes, because elderly patients are generally unable to tolerate intensive treatment. Three of the panelists commented that elderly patients are likely to have shorter survival than younger patients because they have more comorbidities; two experts agreed that comorbidities have a direct effect on complications and symptoms. Agreement was reached on the association between age and QoL. The expert who did not agree that age affected QoL commented that elderly patients were more likely to have comorbidities than younger patients, which would reduce QoL.

The disease process was separated into disease activity, and complications and symptoms. There was consensus for the factors included in each of these groups and the relationships between them: disease activity affected complications and symptoms; complications affected symptoms. Consensus was also reached that disease activity, comorbidities, and complications affect OS, and that age, comorbidities, and complications and symptoms affect QoL. The physician-validated conceptual model is shown in Figure 5.

### Exploratory Analyses to Quantify the Conceptual Model for Economic Modeling Using Real-World Data From the Czech RMG

Exploratory analyses used data from adults enrolled in the Czech RMG who had been diagnosed with symptomatic MM between May 2007 and April 2016; 3,027 patients with newly diagnosed MM were included in the analysis. Median OS from diagnosis was 49.9 months (95% confidence interval = 46.2–53.7) and median follow-up (estimated using the one-minus-survival curve for a slightly smaller sample) was approximately 50 months.

Pairwise correlation analysis was performed on MM attributes at diagnosis to identify which attributes were correlated with each other (Table 2). Strong positive correlations were identified between pain and ECOG performance status, extramedullary disease and bone lesions, and between bone lesions and fatigue (all *P* < 0.001). Negative correlations were identified between several factors, including between anemia and fatigue (*P* < 0.001), which is not surprising because increased hemoglobin reduces fatigue. This negative correlation was observed in all treatment lines, although this may be because the available data on fatigue were reported as a toxicity rather than as a symptom. In general, the correlations identified were in agreement with the outcomes of the Delphi panel but with some exceptions. The panel noted positive correlations between anemia and infection, and between age and anemia, whereas statistically significant negative correlations between these attributes were identified using the RMG data. The Delphi panel also agreed that age was positively associated with anemia and infection, whereas these associations were not found to be statistically significant in the correlation analysis (data not shown). Not all of the attributes and their relationships identified by the Delphi panel could be quantified because of insufficient data in the RMG.

**Table 2:** Pairwise Analysis of Correlations Between Multiple Myeloma Attributes at Diagnosis^a^

	Attribute 1	Correlation (*R*)	Attribute 2
Patient characteristics	ECOG performance status	0.120; *P* < 0.001	Age
		0.066; *P* < 0.001	Extramedullary Mass
		0.083; *P* < 0.001	Extramedullary Disease: Count
		0.064; *P* = 0.002	Kappa FLC level
		0.065; *P* = 0.001	Bone Marrow Plasma Count
		0.09; *P* < 0.001	Serum LDH Level
		−0.258; *P* < 0.001	Albumin Level
		0.22; *P* < 0.001	β_2_ Microglobulin Level
		0.112; *P* < 0.001	Hypercalcemia
		−0.192; *P* < 0.001	Anemia
		0.154; *P* < 0.001	Renal Complications
		0.170; *P* < 0.001	Bone Lesions
		−0.068; *P =* 0.002	Neutropenia
		0.175; *P* < 0.001	Pain
		0.059; *P =* 0.001	Fatigue
		0.095; *P* < 0.001	Infections
	Age	0.121; *P* = 0.001	Hyperdiploidy
		−0.098; *P* < 0.001	Extramedullary Mass
		−0.084; *P* < 0.001	Extramedullary Disease: Count
		0.063; *P* = 0.002	Lambda FLC level
		−0.144; *P* < 0.001	Albumin Level
		0.198; *P* < 0.001	β_2_ Microglobulin Level
		−0.09; *P* < 0.001	Hypercalcemia
		−0.153; *P* < 0.001	Anemia
		0.071; *P* < 0.001	Renal Complications
		−0.057; *P* = 0.003	Bone Lesions
		−0.083; *P* < 0.001	Pain
		0.06; *P* = 0.001	Fatigue
Genetic factors (at diagnosis)	del(17p)	0.108; *P* = 0.037	t(4;14)
		0.099; *P* = 0.007	del(13)(q14)/monosomy 13
		−0.081; *P* = 0.025	M Protein Level
		0.081; *P* = 0.037	Kappa FLC Level
		0.115; *P* = 0.001	Hypercalcemia
		−0.082; *P* = 0.030	Nervous System
		0.071; *P* = 0.045	Bone Fractures
	t(11;14)	−0.156; *P =* 0.026	t(4;14)
		−0.177; *P =* 0.004	M Protein Level
		0.119; *P =* 0.048	Bone Fractures
	t(4;14)	0.305; *P* < 0.001	del(13)(q14)/monosomy 13
		0.294; *P* < 0.001	M Protein Level
		0.217; *P* < 0.001	Bone Marrow Plasma Count
		−0.296; *P* < 0.001	Albumin Level
		0.113; *P* = 0.022	β_2_ Microglobulin Level
		−0.182; *P* < 0.001	Anemia
		0.132; *P* = 0.007	Fatigue
	t(14;16)	0.116; *P* = 0.006	del(13)(q14)/monosomy 13
	del(13)(q14)/monosomy 13	−0.172; *P* < 0.001	Hyperdiploidy
		0.069; *P* = 0.044	β_2_ Microglobulin Level
		0.084; *P* = 0.013	Hypercalcemia
		−0.073; *P* = 0.030	Anemia
		0.097; *P* = 0.004	Renal Complications
		−0.084; *P* = 0.023	Infections
	Hyperdiploidy	−0.076; *P* = 0.042	Albumin Level
		−0.104; *P* = 0.005	Anemia
		−0.111; *P* = 0.005	Nervous System
Disease characteristics	M Protein Level	−0.089; *P* < 0.001	Extramedullary Mass
		−0.07; *P* < 0.001	Extramedullary Disease: Count
		−0.110; *P* < 0.001	Lambda FLC Level
		−0.050; *P* = 0.019	Kappa FLC Level
		0.255; *P* < 0.001	Bone Marrow Plasma Count
		−0.240; *P* < 0.001	Serum LDH Level
		−0.527; *P* < 0.001	Albumin Level
		0.167; *P* < 0.001	β_2_ Microglobulin Level
		−0.066; *P* < 0.001	Hypercalcemia
		−0.338; *P* < 0.001	Anemia
		−0.049; *P* = 0.009	Renal Complications
		0.145; *P* < 0.001	Neutropenia
		0.041; *P* = 0.028	Pain
		0.155; *P* < 0.001	Fatigue
		0.058; *P* = 0.008	Infections
	Extramedullary Mass	0.861; *P* < 0.001	Extramedullary Disease: Count
		−0.095; *P* < 0.001	Bone Marrow Plasma Count
		0.053; *P* = 0.004	Albumin Level
		−0.102; *P* < 0.001	β_2_ Microglobulin Level
		0.150; *P* < 0.001	Anemia
		−0.080; *P* < 0.001	Renal Complications
		0.133; *P* < 0.001	Bone Lesions
		0.195; *P* < 0.001	Pain
		−0.084; *P* < 0.001	Fatigue
	Extramedullary Disease: Count	−0.075; *P* < 0.001	Bone Marrow Plasma Count
		0.045; *P* = 0.019	Serum LDH Level
		−0.065; *P* = 0.001	β_2_ Microglobulin Level
		0.120; *P* < 0.001	Anemia
		−0.061; *P* = 0.001	Renal Complications
		0.050; *P* = 0.019	Nervous System
		0.122; *P* < 0.001	Bone Lesions
		−0.056; *P* = 0.010	Neutropenia
		0.159; *P* < 0.001	Pain
		−0.069; *P* < 0.001	Fatigue
		0.038; *P* = 0.041	Bone Fractures
	Lambda FLC Level	−0.535; *P* < 0.001	Kappa FLC level
		−0.853; *P* < 0.001	Kappa/Lambda FLC Ratio
		−0.044; *P* = 0.042	Bone Marrow Plasma Count
		0.073; *P* = 0.001	Serum LDH Level
		−0.045; *P* = 0.031	Albumin Level
		0.217; *P* < 0.001	β_2_ Microglobulin Level
		−0.067; *P* = 0.001	Anemia
		0.235; *P* < 0.001	Renal Complications
		−0.105; *P* < 0.001	Bone Lesions
		−0.094; *P* < 0.001	Pain
		0.083; *P* < 0.001	Fatigue
	Kappa FLC Level	0.879; *P* < 0.001	Kappa/Lambda FLC Ratio
		0.115; *P* < 0.001	Bone Marrow Plasma Count
		0.067; *P* = 0.002	Serum LDH Level
		0.068; *P* = 0.001	Albumin Level
		0.208; *P* < 0.001	β_2_ Microglobulin Level
		0.096; *P* < 0.001	Hypercalcemia
		−0.121; *P* < 0.001	Anemia
		0.210; *P* < 0.001	Renal Complications
		0.077; *P* = 0.001	Bone Lesions
		0.088; *P* < 0.001	Pain
		0.054; *P* = 0.009	Fatigue
	Kappa/Lambda FLC Ratio	0.112; *P* < 0.001	Bone Marrow Plasma Count
		0.062; *P* = 0.003	Albumin Level
		0.070; *P* = 0.001	Hypercalcemia
		−0.045; *P* = 0.034	Anemia
		0.107; *P* < 0.001	Bone Lesions
		0.113; *P* < 0.001	Pain
	Bone Marrow Plasma Count	0.054; *P* = 0.006	Serum LDH level
		−0.130; *P* < 0.001	Albumin Level
		0.310; *P* < 0.001	β_2_ Microglobulin Level
		0.135; *P* < 0.001	Hypercalcemia
		−0.361; *P* < 0.001	Anemia
		0.146; *P* < 0.001	Renal Complications
		0.087; *P* < 0.001	Bone Lesions
		0.152; *P* < 0.001	Neutropenia
		0.104; *P* < 0.001	Pain
		0.167; *P* < 0.001	Fatigue
		0.084; *P* < 0.001	Infections
	Serum LDH Level	0.066; *P* = 0.001	Albumin Level
		0.096; *P* < 0.001	β_2_ Microglobulin Level
		0.129; *P* < 0.001	Renal Complications
		0.064; *P* = 0.004	Infections
	Albumin Level	−0.313; *P* < 0.001	β_2_ Microglobulin Level
		0.131; *P* < 0.001	Hypercalcemia
		0.403; *P* < 0.001	Anemia
		−0.133; *P* < 0.001	Renal Complications
		0.048; *P* = 0.023	Nervous System
		−0.19; *P* < 0.001	Fatigue
		−0.097; *P* < 0.001	Infections
		−0.048; *P* = 0.009	Bone fractures
	β_2_ Microglobulin Level	0.152; *P* < 0.001	Hypercalcemia
		−0.537; *P* < 0.001	Anemia
		0.730; *P* < 0.001	Renal Complications
		0.059; *P* = 0.007	Neutropenia
		0.049; *P* = 0.010	Pain
		0.323; *P* < 0.001	Fatigue
		0.137; *P* < 0.001	Infections
Complications	Hypercalcemia	0.222; *P* < 0.001	Renal Complications
		0.156; *P* < 0.001	Bone Lesions
		0.180; *P* < 0.001	Pain
		0.044; *P* = 0.041	Infections
		0.058; *P* = 0.002	Bone Fractures
	Anemia	−0.376; *P* < 0.001	Renal Complications
		−0.146; *P* < 0.001	Neutropenia
		−0.506; *P* < 0.001	Fatigue
		−0.106; *P* < 0.001	Infections
	Renal Complications	0.260; *P* < 0.001	Fatigue
		0.105; *P* < 0.001	Infections
	Nervous System	0.134; *P* < 0.001	Neutropenia
		0.115; *P* < 0.001	Fatigue
		0.102; *P* < 0.001	Infections
	Bone Lesions	0.740; *P* < 0.001	Pain
		−0.039; *P* = 0.040	Fatigue
	Neutropenia	0.059; *P* = 0.006	Pain
		0.087; *P* < 0.001	Fatigue
		0.121; *P* < 0.001	Infections
	Pain	0.046; *P* = 0.031	Infections
		0.054; *P* = 0.003	Bone Fractures
	Fatigue	0.099; *P* < 0.001	Infections

ECOG, European Cooperative Oncology Group; FLC, free light chain; LDH, lactate dehydrogenase.

a Only correlations that reached statistical significance are presented in the table. Each correlation is presented once only to avoid repetition. Proxies were used for some attributes. Anemia: hemoglobin; hypercalcemia: calcium; renal complications: creatinine; nervous system; grade of neuropathy; pain: presence of at least two osteolytic lesions or a bone-related extramedullary mass; numbness and tingling: neuropathy; fatigue and infection: toxicity. Pearson’s *R* correlation coefficients were calculated and statistical significance was set at *P* < 0.05.

In the first and second treatment lines, significant positive correlations were identified between age and ECOG performance status, and between β_2_ microglobulin level and fatigue. Bone lesions and pain were also significantly positively correlated with each other (Online Appendix 1; Table S9). The strength of the correlations tended to change with successive relapses. For example, the strengths of the positive correlations between β_2_ microglobulin level and fatigue, and between renal complications and fatigue, decreased from the first to the fourth treatment line (data not shown). The variation in correlation strength probably reflects variation in patient characteristics across lines: patients who survive and receive a subsequent line of treatment are likely to have different attributes from those who died or did not (yet) progress to the next treatment line.

**Figure 6:**
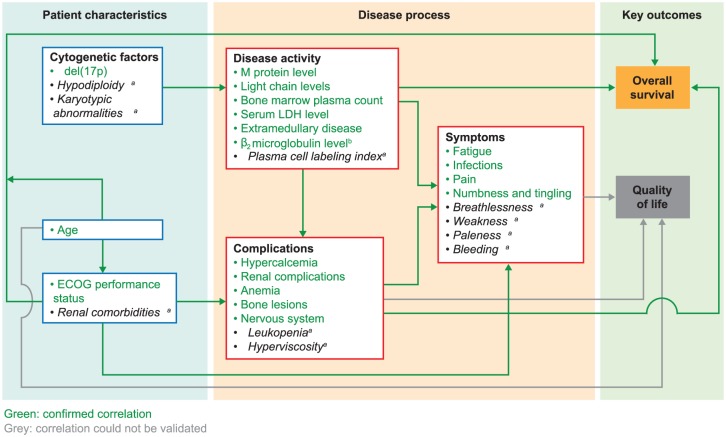
Final conceptual model of multiple myeloma for economic modeling. ECOG, Eastern Cooperative Oncology Group; ISS, International Staging System; LDH, lactate dehydrogenase; RMG, Registry of Monoclonal Gammopathies. The model was refined and finalized using input from the Delphi panel and the pairwise analysis and Cox regression analysis of real-world data from the Czech RMG. ^a^Correlation not confirmed because data unavailable in RMG dataset. ^b^Estimated from ISS stage at diagnosis.

Multiple Cox regression analysis using data from the RMG confirmed several significant predictors of OS in our conceptual model, including age and ECOG performance status. Disease factors such as extramedullary disease, ISS stage, revised ISS (R-ISS) stage at diagnosis, thrombocyte count, and levels of creatinine were also confirmed to affect OS significantly (Table 3).^28^ Levels of serum LDH also significantly affected OS, and even though consensus was not reached by the Delphi panel, this was included in the model because of its relevance as part of the R-ISS. Calcium level, bone lesions, and bone marrow plasma count were not independent predictors of OS; however, these attributes were significantly positively correlated with other attributes in the univariate analysis (Table 2), meaning that they may indirectly influence OS. Thus, these attributes were deemed to be important features of the model (supported by evidence from the literature searches and Delphi panel) and were included in the final conceptual model (Figure 6). QoL data were lacking in the RMG; so although many attributes were considered to affect QoL, these associations could not be quantified (Figure 6). Similarly, several attributes, including hypodiploidy, karyotypic abnormalities, renal comorbidities, symptoms, and plasma cell labeling index, could not be validated statistically but were all deemed by the Delphi panel to affect the disease process and patient outcomes and were therefore included in the final conceptual model (Figure 6).

**Table 3:** Multiple Cox Regression Analysis of Predictors of OS at Initiation of First Treatment Line

Predictor	HR for Death (95% CI)	
	Full Model	Selected Predictors^a^
Age at diagnosis, Years		
65–75 v. <65	1.42 (1.24–1.62)***	1.41 (1.24–1.61)***
>75 v. <65	2.11 (1.82–2.45)***	2.10 (1.81–2.43)***
ECOG performance status		
1–2 v. 0	1.33 (1.03–1.71)*	1.33 (1.03–1.71)*
3–4 v. 0	2.26 (1.65–3.09)***	2.25 (1.65–3.06)***
LDH level, U/L		
>360 v. ≤360	1.68 (1.29–2.19)***	1.73 (1.33–2.25)***
R-ISS stage at diagnosis^b^		
II v. I	1.98 (1.03–3.80)*	2.02 (1.06–3.89)*
III v. I	2.26 (1.16–4.42)*	2.33 (1.19–4.54)*
ISS stage at diagnosis		
II v. I	1.57 (1.27–1.94)***	1.61 (1.31–1.99)***
III v. I	1.98 (1.58–2.48)***	2.04 (1.63–2.55)***
Creatinine level, mmol/L^c^		
>173 v. ≤173	1.37 (1.15–1.62)***	1.35 (1.14–1.59)***
Extramedullary disease		
Yes v. No/NA	1.45 (1.16–1.83)**	1.46 (1.16–1.83)**
Thrombocyte count, 10^9^/L		
≤100 v. >100	1.88 (1.51–2.34)***	1.86 (1.50–2.32)***
Calcium level, mmol/L^c^		
>2.75 v. ≤2.75	0.90 (0.72–1.13)	—
Bone lesions^d^		
Yes v. No	1.10 (0.91–1.33)	—
Bone marrow plasma cell count, %		
20–70 v. <20	1.16 (1.00–1.35)	—
>70 v. <20	1.28 (0.96–1.71)	—

CI, confidence interval; CRAB, hypercalcemia, renal insufficiency, anemia, and lytic bone lesions or osteoporosis; CT, computed tomography; ECOG, European Cooperative Oncology Group; HR, hazard ratio; ISS, International Staging System; LDH, lactate dehydrogenase; NA, not available; OS, overall survival; PET, positron emission tomography; R-ISS, Revised International Staging System.

a Backward selection was performed using Akaike’s information criterion.

b R-ISS stage is a validated composite measure of risk which includes ISS, CA, and LDH and hence was included in the Cox model.

c Cutoff levels derived from CRAB-related reasons for initiating therapy.

d Evaluated by different techniques (X-ray, nuclear magnetic resonance, CT, PET, PET/CT, or methoxy-isobutyl-isonitrile imaging).

Significance level set at *P* < 0.05. *P < 0.05; **P < 0.01; ***P < 0.001.

## Discussion

In line with the recommendations of the ISPOR–SMDM Task Force,^20,22^ the conceptual model described here was developed for use in clinical decision making and health economic modeling. This is the first conceptual model of MM to incorporate the relevance of disease and patient attributes to disease progression and patient outcomes.

A deep understanding of the current evidence base for MM was gained by systematic and targeted literature reviews. The draft conceptual model was based on variables identified from the literature reviews and was refined using the Delphi method. A conceptual model for use in clinical decision making and economic modeling requires evidence of its relevance to the disease setting in clinical practice. However, the lack of consensus on the associations between some attributes and outcomes shows that current understanding of how aspects of MM affect disease progression and patient outcomes differs among clinicians, and is based on experience rather than evidence. This is also reflected in the limited data from randomized clinical trials on how certain attributes affect the disease process and patient outcomes. As a result, potential causal relationships in our conceptual model were identified through the insight and experience of the MM experts. Statistical analyses of real-world data from the Czech RMG corroborated many of these associations.

The Delphi panel deemed disease activity to be central to the conceptual model, affecting complications, symptoms, OS, and QoL. The panel agreed that age influenced ECOG performance status and QoL, and the correlation between age and ECOG performance status was confirmed using data from the RMG; the correlation between age and QoL could not be confirmed because there were no relevant data in the RMG. The Delphi panel agreed that age may influence OS indirectly, particularly via comorbidities: older patients are more likely than younger patients to have comorbidities,^29^ and a study of patients older than 65 years with newly diagnosed MM found that higher Charlson Comorbidity Indices were associated with significantly shorter OS.^30^ A retrospective European patient chart review showed that patients with comorbidities (including anemia, low serum albumin, and neutropenia) and adverse events were significantly less likely to continue treatment than those without such comorbidities (*P* < 0.05).^31^

In line with the Delphi panel, the analysis of clinical practice data from the Czech RMG confirmed age as a predictor of OS. In another study, age directly affected OS independently of comorbidities: survival is significantly shorter in older than in younger patients with comorbidities.^32^ As patients age, they become increasingly frail and OS is worse than in fit patients.^33^ The influence of age on OS may also be due to differences in the management of older and younger patients with MM: high-dose chemotherapy followed by autologous SCT is the standard of care for younger patients, whereas those older than 65 years are likely to receive chemotherapy or targeted agents.^34,35^ Data from a retrospective chart review across seven European countries found that patients older than 65 years were less likely than younger patients to receive autologous SCT (21% v. 79%).^6^ Two randomized clinical trials have shown that high-dose chemotherapy followed by autologous SCT is associated with an improved response rate, event-free survival, and OS compared with conventional chemotherapy.^36,37^ In addition, the shorter OS in older patients may be due to discontinuation of treatment. Another retrospective European chart review of patients with MM found that individuals older than 75 years were significantly less likely than younger patients to continue treatment (*P* < 0.0001).^31^

There was some disagreement among the Delphi panel about the influence of age on symptoms and complications. Only two of the experts agreed that age might influence certain complications, such as neuropathy, but not others. This is in line with published data in which the link between age and neuropathy is unclear.^38–40^ Pairwise comparisons of clinical data in our study identified correlations between age and symptoms only in patients receiving third- or fourth-line treatment. Furthermore, age was found to be negatively associated with neuropathy only in the third treatment line, and not in any other line.

Our panel also found that complications and symptoms influenced QoL, although insufficient data were available for these relationships to be validated statistically. A conceptual model developed by others focusing solely on the impact of MM and its treatment on QoL found that the burden of MM symptoms and treatment negatively affected QoL.^21^ However, their QoL conceptual model differs from the model developed here, which is the first to conceptualize the MM disease process for use as a predictive tool in the economic evaluation of health care interventions.

MM is a heterogeneous disease and practice patterns vary, which may affect patient outcomes. Data from a European patient chart review found that patients who were healthier at baseline were more likely to have received autologous SCT than those in poor health or with a high ECOG performance status score.^6^ Furthermore, differences between guidelines in the diagnostic criteria and the influence of cytogenetic factors on prognosis, for example, may also result in varied approaches to the management of patients with MM. The International Myeloma Working Group consensus for the treatment of patients with high-risk cytogenetics recommends different treatment strategies according to the specific cytogenetic abnormalities.^41^ Guidelines may make recommendations based on data from retrospective cohort studies or non-controlled trials in some instances.^34,35,42,43^ Therefore, consensus opinions based on clinical experience, such as those defined here, may be valuable to clinicians when developing guideline recommendations and making treatment decisions. We validated and refined the model using clinical data; therefore, our conceptual model may be used for clinical decision making and health economic modeling.

This study has some limitations. Consensus reached during the Delphi method represents the opinion of the participants and may not necessarily represent the opinions of clinicians in general. Additionally, our panel was small, and members were all practicing in Europe and therefore provided their input based on clinical practice in this region only. “Agreement” was defined as at least 50% of the panel members sharing the same opinion. By definition, this meant that the other two members may not have shared this opinion (although the other two members must have had different opinions from each other). Attributes and associations for which only 50% of panel members agreed may be weaker than those for which more members agreed. Another limitation is that, for congress abstracts identified, data from related posters or oral presentations were not obtained, meaning that some relevant information may not have been included. Clinical data were not always available because MM is a rare disease, so some parameters included in the model could not be validated statistically. For some attributes, proxy measures were used. For example, the presence of two or more osteolytic lesions or a bone-related extramedullary mass were used as proxies for pain, and numbness and tingling were used as indicators for neuropathy. Toxicity was used as a proxy for infection and fatigue; however, other factors could cause these symptoms. Data entered into registries may not be complete, and the approach to recording information may vary among physicians. Data on cytogenetic risk are limited: these data are missing for many patients in the RMG and data on comorbidities were not available. Nonetheless, most of the parameters that were included did have corresponding real-world data and the interrelationships between attributes and groups could be verified. In addition, the cutoff for determining high LDH levels may differ among laboratories, which could introduce variability (random error) in the analysis.

The predictors of OS were validated only with respect to their direct influence on OS; multivariate analyses to investigate how other attributes influence each other (e.g., how cytogenetic factors affect M protein) were not conducted. Furthermore, the lack of QoL data in the Czech RMG meant that it was not possible to validate the many attributes in the model considered to affect QoL. In addition, the conceptual model figure indicates that more variables are associated with OS than are available in the RMG data. We were therefore unable to test whether these variables confound the associations in the multivariable Cox models, which means that the associations in the Cox models are prone to residual confounding bias. The multivariable analyses should therefore be regarded as exploratory. MM is a complicated and progressive disease and the model does not consider how disease activity leading to complications could affect OS; therefore, there is scope for future refinement. Although our model considers only the disease process and patient outcomes, treatment could also be incorporated. Treatment would be expected to have a direct effect on disease activity and an indirect effect on complications and symptoms. The model could be further refined by including healthcare resource utilization (HRU) during management of MM. Additionally, treatment-related adverse events could also be considered, and may have effects on HRU, QoL, and OS. Including treatment effect in the model could allow the impact of sequential treatments on the disease process to be assessed. Further research is required to explore this. Real-world data, such as those from patient chart studies and disease registries, provide valuable information on treatment patterns and patient outcomes,^6,24,27,31^ and can be used to quantify the impact of therapeutic interventions on attributes and their interactions within the model. These data also provide the potential to examine the effects on outcomes of changes in patient characteristics over time. Additionally, there is a need for further research on how the interrelationships between attributes change over time.

## Conclusions

This is the first conceptual model of MM to provide a systematic representation of the interrelationship between patient characteristics and disease processes on key outcomes. This model has been validated using a Delphi panel of experts and real-world evidence and is therefore appropriate for use in clinical decision making and in economic modeling. Furthermore, it provides a framework to guide further research on the impact of treatment on patient outcomes over time. This will allow researchers to quantify and to qualify the effect that disease attributes have on aspects of the disease process and will help predict disease progression and patient outcomes, furthering our understanding of the underlying disease process in MM and how specific therapeutic interventions can benefit patients.

## Supplemental Material

DS_10.1177_2381468318814253 – Supplemental material for Development of an Initial Conceptual Model of Multiple Myeloma to Support Clinical and Health Economics Decision Making Click here for additional data file. Supplemental material, DS_10.1177_2381468318814253 for Development of an Initial Conceptual Model of Multiple Myeloma to Support Clinical and Health Economics Decision Making by Sebastian Gonzalez-McQuire, Meletios-Athanassios Dimopoulos, Katja Weisel, Walter Bouwmeester, Roman Hájek, Marco Campioni, Craig Bennison, Weiwei Xu, Krystallia Pantiri, Marja Hensen, Evangelos Terpos and Stefan Knop in MDM Policy & Practice
