# Supplementary material for: Development of an Initial Conceptual Model of Multiple Myeloma to Support Clinical and Health Economics Decision Making
Source: MDM Policy Pract. 2019 Jan 17;4(1):2381468318814253. doi: 10.1177/2381468318814253 (PMC6350154; doi:10.1177/2381468318814253)
Supplement: DS_10.1177_2381468318814253 – Supplemental material for Development of an Initial Conceptual Model of Multiple Myeloma to Support Clinical and Health Economics Decision Making [file DS_10.1177_2381468318814253.pdf]

Appendix 1

Table S1 Databases Searched

**Conceptual Model**	**HE Models**	**HTA Reports**	**Guidelines**
Embase (using ProQuest)	Embase (using ProQuest)	National Institute for Health and Care Excellence (NICE)	Guidelines International Library
Medline (using ProQuest)	Medline (using ProQuest)	Scottish Medicines Consortium (SMC)	Standards and Guidelines Evidence (SAGE)
American Society of Hematology (ASH) annual meeting proceedings	ASH abstracts (using ProQuest)	Haute Autorité de Santé (HAS)	National Comprehensive Cancer Network (NCCN)
American Society of Clinical Oncology (ASCO) annual meeting proceedings	American Society of Clinical Oncology (ASCO) annual meeting proceedings	Gemeinsamer Bundesausschuss (G-BA)	NIH National Cancer institute
		Institut für Qualität und Wirtschaftlichkeit im Gesundheitswesen (IQWiG)	National Guideline Clearinghouse (FDA database)
		Tandvårds- och läkemedelsförmånsverket (TLV)	European Society for Medical Oncology (ESMO)
		Zorginstituut Nederland	National Institute for Health and Care Excellence (NICE)
		Department of Health Australia, Review of Health Technology Assessments (HTA)	Scottish Intercollegiate Guidelines Network (SIGN)
		Canadian Agency for Drugs and Technologies in Health (CADTH)	Deutsche Gesellschaft fur Hamatologie und medizinische Onkologie (DGHO)- Onkopedia
		Centre of Reviews and Disseminations (CRD)	

Table S2 Search Terms used for the Systematic ProQuest Search of Embase and Medline Databases: Conceptual Models

Multiple Myeloma	1	TI,AB(“multiple myeloma”) OR EMB.EXACT("multiple myeloma") OR MESH.EXACT("Multiple Myeloma")	99,295
Conceptual Model	2	#1 AND (TI,AB("conceptual model" OR "conceptual framework" OR "regression model" OR "endpoint model" OR "disease model" OR (disease AND (association OR correlation OR relationship OR attribute))) OR EMB.EXACT.EXPLODE("conceptual framework"))	3,324
Limits	3	#2 AND (LA(English) AND PD(2004-2014))	1,985
**Total**			**1,985**

The same search terms were used to search the American Society of Hematology library (2012–2014).

Table S3 Search Terms used for Systematic Review of the American Society of Clinical Oncology Library

Search in meeting library → Abstracts			
Multiple Myeloma	1	Keywords: multiple myeloma	539
2014	2	2014 ASCO Annual meeting	85
2013	3	2013 ASCO Annual meeting	93
2012	4	2012 ASCO Annual meeting	101
**Total**			**279**

Note: ASCO = American Society of Clinical Oncology.

Table S4 Search Terms used for the Systematic ProQuest Search of Embase and Medline Databases: Health Economic Models

Multiple Myeloma	1	TI,AB(“multiple myeloma”) OR EMB.EXACT("multiple myeloma") OR MESH.EXACT("Multiple Myeloma")	99,295
Health Economic	2	#1 AND (TI,AB("health economics" OR "economic evaluation" OR pharmacoeconomic OR economic*model OR cost*effectiveness OR cost*benefit OR cost*utility* OR cost*minimi*ation OR "cost comparison" OR "ICER" OR "willingness-to-pay" OR "willingness to pay") OR (EMB.EXACT.EXPLODE("economic evaluation") OR EMB.EXACT("statistical model") OR EMB.EXACT("pharmacoeconomics")) OR (MESH.EXACT.EXPLODE("Costs and Cost Analysis") OR MESH.EXACT("Models, Economic") OR MESH.EXACT("Economics, Pharmaceutical")))	857
Limits	3	#2 AND (LA(English) AND PD(2004-2014))	661
**Total**			**661**

Table S5 Search Terms for Health Technology Assessment Reports

**National Institute for Health and Care Excellence**			
Disease	1	Multiple myeloma	42
Filter 1	2	Guidance	18
Filter 2	3	Technology appraisals	13
**Total**			**13**
**Scottish Medicines Consortium**			
On the homepage, enter the term ‘’multiple myeloma’’ in the search engine. Under ‘’SMC advice’’ section (125 hits).			
**Haute Autorité de Santé**			
Disease	1	Myeloma	3
	2	Multiple myeloma	3
Filter 1	3	Avis et evaluation (Opinions and assessments)	3
**Total**			**3**
**Gemeinsamer Bundesausschuss**			
Disease	1	Myelom	64
Filter by content type	2	Nutzenbewertung (evaluation)	12
**Total**			**12**
**Institut für Qualität und Wirtschaftlichkeit im Gesundheitswesen**			
Disease	1	Multiple Myelom	62
Filter by content type	2	Dossierbewertung (Dossier assessment)	2
**Total**			**2**
**Tandvårds- och läkemedelsförmånsverket**			
Disease	1	Multipelt myelom	40
Filter by content type	2	Läkemedel (drugs)	2
**Total**			**2**
**Zorginstituut Nederland**			
Disease	1	Myeloma	8
	2	Multiple Myeloma	8

Filter	3	Geneesmiddelenbeoordelingen	7
**Total**			**7**
**Department of Health Australia**			
Disease	1	Multiple Myeloma	1,211
	2	HTA reports for Multiple Myeloma	296
**Total**			**296**
**Canadian Agency for Drugs and Technologies in Health**			
Disease	1	Multiple myeloma	195
Filter by publication type	2	Publication type	61
	3	Technology reports	9
	4	Technology overviews	3
	5	Therapeutic review	1
**Total**	**6**	**3 AND 4 AND 5**	**13**
**Centre of Reviews and Disseminations**			
Disease	1	Multiple myeloma	191
Filter by content type	2	NHS EED and HTA	112
**Total**			**112**

Note: HTA = health technology appraisail; NHS EED = National Health Service Economic Evaluation Database.

Table S6 Search Terms for Guidelines

Guidelines International Library

On the homepage, enter the term “myeloma” in the search engine and filter by guidelines (12 hits).

SAGE

On the homepage of SAGE, enter the term “myeloma” in the search engine (1 hit).

NCCN

At the NCCN Homepage, in the menu bar, select NCCN Guidelines → NCCN guidelines for treatment of cancer by site → multiple myeloma (1 hit).

NIH National Cancer Institute

At the homepage, under “Cancer topics”, select “treatment”, under “treatment for specific cancer”, select “PDQ® Cancer Information Summaries: Adult Treatment”, in the “Alphabetical List of PDQ® Adult Cancer Treatment Summaries” that appears, find the title “Multiple Myeloma and Other Plasma Cell Neoplasms” and select “Health professional”. On the menu on the left side of the webpage, select “Treatment for Multiple Myeloma” (1 hit).

European Society for Medical Oncology

At the Homepage, in the Menu on the top of the webpage, select “Guidelines and Practice” → Clinical practice guidelines → under “Title”, select “Haematologic malignancies” → Latest enhanced and revised set of guidelines → Multiple myeloma (1 hits).

Scottish Intercollegiate Guidelines Network

In the menu at the left side of the homepage, select “Guidelines” → Published guidelines by topic → Cancer (24 hits).

Deutsche Gesellschaft für Hämatologie und Medizinische Onkologie

At the homepage select “onkopedia leitlinien”, enter the term “myelom” in the search engine → 13 hits or “multiple* myelom” (9 hits).

Topic	Search #	Search Terms	Number of Hits
**National Guideline Clearinghouse (FDA Database)**			
Disease	1	Multiple myeloma	42
**Total**			**42**
**National Institute for Health and Care Excellence**			
Disease	1	Multiple myeloma	42
Filter 1	2	Guidance	18
Filter 2	3	Guideline	4
**Total**			**4**

Notes: FDA = Food and Drug Administration; NCCN = National Comprehensive Cancer Network; NIH = National Institutes of Health; PDQ = Physician Data Query; SAGE = Standards and Guidelines Evidence.

Table S7 Literature Review: Inclusion Criteria

	Conceptual models	HE models	HTA reports	Guidelines
Publication year	2004–2014	2004–2014	2004–2014	2009–2014
Language	English	English	English; local language if English is not available	English; local language if English is not available
Indication	Multiple myeloma population/patients			
Topic / content	Include: Publications concerning conceptual models, disease models, data on relationships, associations, correlations, attributes, adverse effects, symptoms, natural history and prognosis for multiple myeloma<br><br>Exclude: Publications with no data on the included topics; studies reporting only the efficacy of therapeutic interventions	Include: HE models concerning pharmaceutical interventions<br><br>Exclude: Publications without HE models; HE models concerning non-pharmaceutical interventions	Include: HTA reports of pharmaceutical interventions<br><br>Exclude: Non HTA reports; HTA reports discussing non-pharmaceutical treatments	Include: Guideline for overall treatment/management of multiple myeloma; one key guideline per country<br><br>Exclude: Documents which are not guidelines; guidelines discussing one pharmaceutical intervention; guidelines on other topics than disease management

HE = health economic; HTA = health technology assessment.

Table S8 Data Extracted from the Literature Searches

- Attributes
- Attribute Influences
- Type of Relationship/Influence of Attribute on other Attributes
- Strength of Relationship
- Significance of Relationship
- Direct or Indirect Relationship
- Type of Model (Qualitative/Quantitative)
- Whether or not Time Influenced the Relationship between Attributes
- Attribute Category (e.g. Patient/Disease Characteristics)
- Attributes per Category
- Attribute Category Influences

Table S9 Pairwise Analysis of Correlations between MM Attributes in the First- and Second-Treatment Lines

First Line

	Attribute 1	Correlation (R)	Attribute 2
**Patient Characteristics**	ECOG Performance Status	0.128; $P < 0.001$	Age
		−0.232; $P < 0.001$	Time Since Diagnosis
		0.110; $P < 0.001$	Extramedullary Mass
		0.113; $P < 0.001$	Extramedullary Disease: Count
		0.063; $P = 0.004$	Bone Marrow Plasma Count
		0.096; $P < 0.001$	Serum LDH Level
		−0.287; $P < 0.001$	Albumin Level
		0.243; $P < 0.001$	β_2 Microglobulin Level
		0.054; $P = 0.010$	Hypercalcemia
		−0.215; $P < 0.001$	Anemia
		0.168; $P < 0.001$	Renal Complications
		0.116; $P < 0.001$	Bone Lesions
		−0.058; $P = 0.008$	Neutropenia
		0.122; $P < 0.001$	Pain
		0.108; $P < 0.001$	Fatigue
		0.098; $P < 0.001$	Infections
	Age	0.039; $P = 0.031$	Time Since Diagnosis
		0.119; $P = 0.001$	Hyperdiploidy
		−0.091; $P < 0.001$	Extramedullary Mass
		−0.073; $P < 0.001$	Extramedullary Disease: Count
		−0.126; $P < 0.001$	Albumin Level
		0.189; $P < 0.001$	β_2 Microglobulin Level
		−0.090; $P < 0.001$	Hypercalcemia
		−0.115; $P < 0.001$	Anemia
		0.082; $P < 0.001$	Renal Complications
		−0.057; $P = 0.009$	Bone Lesions
		−0.125; $P < 0.001$	Pain
	Time Since Diagnosis	−0.111; $P = 0.023$	t(4;14)
		−0.071; $P = 0.035$	del(13)(q14)/ monosomy 13
		−0.078; $P < 0.001$	M Protein Level
		−0.252; $P < 0.001$	Bone Marrow Plasma Count
		−0.051; $P = 0.018$	Serum LDH Level
		0.173; $P < 0.001$	Albumin Level
		−0.261; $P < 0.001$	β_2 Microglobulin Level
		−0.169; $P < 0.001$	Hypercalcemia
		0.254; $P < 0.001$	Anemia
		−0.265; $P < 0.001$	Renal Complications

		−0.105; $P < 0.001$	Bone Lesions
		−0.053; $P = 0.013$	Neutropenia
		−0.116; $P < 0.001$	Pain
		−0.123; $P < 0.001$	Fatigue
		−0.066; $P = 0.002$	Infections
		−0.043; $P = 0.017$	Bone Fractures
**Genetic Factors (At Diagnosis)**	del(17p)	0.108; $P = 0.037$	t(4;14)
		0.099; $P = 0.007$	del(13)(q14)/ monosomy 13
		−0.094; $P = 0.011$	M Protein Level
		0.107; $P = 0.004$	Hypercalcemia
		−0.082; $P = 0.030$	Nervous System
		0.071; $P = 0.045$	Bone Fractures
	t(11;14)	−0.156; $P = 0.026$	t(4;14)
		−0.187; $P = 0.003$	M Protein Level
		0.119; $P = 0.048$	Bone Fractures
	t(4;14)	0.305; $P < 0.001$	del(13)(q14)/ monosomy 13
		0.324; $P < 0.001$	M Protein Level
		0.212; $P < 0.001$	Bone Marrow Plasma Count
		−0.310; $P < 0.001$	Albumin Level
		0.119; $P = 0.025$	β_2 Microglobulin
		−0.163; $P = 0.002$	Anemia
		0.115; $P = 0.019$	Fatigue
	t(14;16)	0.116; $P = 0.006$	del(13)(q14) /monosomy 13
		0.097; $P = 0.032$	Bone Marrow Plasma Count
		0.107; $P = 0.016$	β_2 Microglobulin Level
	del(13)(q14)/monosomy 13	−0.172; $P < 0.001$	Hyperdiploidy
		−0.090; $P = 0.011$	Anemia
		0.084; $P = 0.018$	Renal Complications
		−0.084; $P = 0.023$	Infections
	Hyperdiploidy	0.096; $P = 0.018$	Bone Marrow Plasma Count
		−0.081; $P = 0.041$	Albumin Level
		−0.149; $P < 0.001$	Anemia
		− 0.111; $P = 0.005$	Nervous System
**Disease Characteristics**	M Protein Level	−0.090; $P < 0.001$	Extramedullary Mass
		−0.074; $P = 0.001$	Extramedullary Disease: Count
		0.058; $P = 0.002$	Kappa/Lambda FLC Ratio
		0.224; $P < 0.001$	Bone Marrow Plasma Count
		−0.252; $P < 0.001$	Serum LDH Level
		−0.508; $P < 0.001$	Albumin Level
		0.082; $P < 0.001$	β_2 Microglobulin

	−0.091; $P < 0.001$	Hypercalcemia
	−0.293; $P < 0.001$	Anemia
	−0.106; $P < 0.001$	Renal Complications
	0.153; $P < 0.001$	Neutropenia
	0.046; $P = 0.016$	Fatigue
	0.057; $P = 0.010$	Infections
	−0.039; $P = 0.040$	Bone Fractures
Extramedullary Mass	−0.088; $P < 0.001$	Bone Marrow Plasma Count
	0.854; $P < 0.001$	Extramedullary Disease: Count
	−0.113; $P < 0.001$	β_2 Microglobulin Level
	0.140; $P < 0.001$	Anemia
	−0.083; $P < 0.001$	Renal Complications
	0.127; $P < 0.001$	Bone Lesions
	−0.051; $P = 0.017$	Neutropenia
	0.190; $P < 0.001$	Pain
	−0.083; $P < 0.001$	Fatigue
Extramedullary Disease: Count	−0.058; $P = 0.008$	Bone Marrow Plasma Count
	−0.069; $P = 0.001$	β_2 Microglobulin Level
	0.101; $P < 0.001$	Anemia
	−0.055; $P = 0.008$	Renal Complications
	0.045; $P = 0.035$	Nervous System
	0.100; $P < 0.001$	Bone Lesions
	−0.064; $P = 0.003$	Neutropenia
	0.155; $P < 0.001$	Pain
	−0.065; $P = 0.002$	Fatigue
Kappa/Lambda FLC Ratio	0.047; $P = 0.032$	Bone Marrow Plasma Count
	−0.056; $P = 0.008$	Renal Complications
	0.087; $P < 0.001$	Bone Lesions
	0.046; $P = 0.012$	Pain
Bone Marrow Plasma Count	0.073; $P = 0.001$	Serum LDH Level
	−0.114; $P < 0.001$	Albumin Level
	0.270; $P < 0.001$	β_2 Microglobulin
	0.118; $P < 0.001$	Hypercalcemia
	−0.366; $P < 0.001$	Anemia
	0.118; $P < 0.001$	Renal Complications
	0.070; $P = 0.002$	Bone lesions
	0.160; $P < 0.001$	Neutropenia
	0.072; $P = 0.001$	Pain
	0.170; $P < 0.001$	Fatigue
	0.079; $P < 0.001$	Infections
Serum LDH Level	0.076; $P < 0.001$	Albumin Level
	0.115; $P < 0.001$	β2 Microglobulin Level
	0.148; $P < 0.001$	Renal Complications
	0.046; $P = 0.032$	Fatigue

		0.051; $P = 0.022$	Infections
	Albumin Level	−0.275; $P < 0.001$	β_2 Microglobulin Level
		0.172; $P < 0.001$	Hypercalcemia
		0.363; $P < 0.001$	Anemia
		−0.123; $P < 0.001$	Renal Complications
		0.043; $P = 0.043$	Nervous System
		−0.172; $P < 0.001$	Fatigue
		−0.081; $P < 0.001$	Infections
		0.053; $P = 0.012$	Bone Fractures
	β_2 Microglobulin Level	0.130; $P < 0.001$	Hypercalcemia
		−0.515; $P < 0.001$	Anemia
		0.736; $P < 0.001$	Renal Complications
		−0.052; $P = 0.023$	Bone Lesions
		0.062; $P = 0.005$	Neutropenia
		0.289; $P < 0.001$	Fatigue
		0.137; $P < 0.001$	Infections
**Complications**	Hypercalcemia	0.192; $P < 0.001$	Renal Complications
		0.120; $P < 0.001$	Bone Lesions
		0.140; $P < 0.001$	Pain
		0.067; $P = 0.001$	Bone Fractures
	Anemia	−0.365; $P < 0.001$	Renal Complications
		0.047; $P = 0.033$	Bone Lesions
		−0.140; $P < 0.001$	Neutropenia
		−0.467; $P < 0.001$	Fatigue
		−0.095; $P < 0.001$	Infections
	Renal Complications	−0.118; $P < 0.001$	Bone Lesions
		−0.082; $P < 0.001$	Pain
		0.229; $P < 0.001$	Fatigue
		0.107; $P < 0.001$	Infections
	Nervous System	0.134; $P < 0.001$	Neutropenia
		0.112; $P < 0.001$	Fatigue
		0.102; $P < 0.001$	Infections
	Bone Lesions	0.063; $P = 0.006$	Neutropenia
		0.675; $P < 0.001$	Pain
		−0.095; $P < 0.001$	Fatigue
	Neutropenia	0.042; $P = 0.050$	Pain
		0.083; $P < 0.001$	Fatigue
		0.121; $P < 0.001$	Infections
	Pain	0.279; $P < 0.001$	Fatigue
		0.050; $P = 0.006$	Bone Fractures
	Fatigue	0.090; $P < 0.001$	Bone Fractures
**Second Line**			
	**Attribute 1**	**Correlation (*R*)**	**Attribute 2**
**Patient Charact eristics**	ECOG Performance Status	0.122; $P < 0.001$	Age
		−0.227; $P < 0.001$	Time Since Diagnosis
		0.061; $P = 0.042$	M Protein Level

		0.189; P < 0.001	Extramedullary Mass
		0.142; P < 0.001	Extramedullary Disease: Counts
		0.105; P = 0.001	Serum LDH Level
		−0.262; P < 0.001	Albumin Level
		0.282; P < 0.001	β_2 Microglobulin Level
		−0.193; P < 0.001	Anemia
		0.160; P < 0.001	Renal Complications
		0.075; P = 0.011	Pain
		0.075; P = 0.010	Fatigue
	Age	0.103; P < 0.001	Time Since Diagnosis
		−0.104; P = 0.037	del(13)(q14)/ monosomy 13
		0.183; P = 0.001	Hyperdiploidy
		0.086; P = 0.003	M Protein Level
		−0.119; P < 0.001	Extramedullary Mass
		−0.139; P < 0.001	Extramedullary Disease: Count
		−0.113; P < 0.001	Albumin Level
		0.169; P < 0.001	β_2 Microglobulin Level
		−0.069; P = 0.017	Hypercalcemia
		−0.085; P = 0.003	Anemia
		0.110; P < 0.001	Renal Complications
		−0.063; P = 0.033	Neutropenia
		−0.110; P < 0.001	Pain
		−0.062; P = 0.037	Infections
	Time Since Diagnosis	−0.189; P < 0.001	M Protein Level
		−0.107; P < 0.001	Extramedullary Mass
		−0.081; P = 0.006	Extramedullary Disease: Count
		−0.121; P = 0.002	Bone Marrow Plasma Count
		−0.091; P = 0.003	Serum LDH Level
		0.270; P < 0.001	Albumin Level
		−0.221; P < 0.001	β_2 Microglobulin Level
		0.087; P = 0.002	Hypercalcemia
		0.290; P < 0.001	Anemia
		−0.104; P < 0.001	Renal Complications
		0.063; P = 0.031	Nervous System
		−0.076; P = 0.010	Neutropenia
		0.058; P = 0.029	Pain
**Genetic factors (At Diagnosis)**	del(17p)	0.202; P = 0.006	t(4;14)
		0.157; P = 0.023	t(14;16)
		0.124; P = 0.04	Serum LDH Level
		0.128; P = 0.017	Bone Fractures
	t(11;14)	−0.237; P = 0.028	t(4;14)

	t(4;14)	0.382; *P* < 0.001	del(13)(q14)/ monosomy 13
		0.220; *P* = 0.003	Extramedullary Disease: Count
	t(14;16)	0.217; *P* = 0.001	del(13)(q14)/ monosomy 13
		−0.165; *P* = 0.007	Pain
	del(13)(q14)/monosomy 13	−0.213; *P* < 0.001	Hyperdiploidy
		0.171; *P* = 0.027	Bone Marrow Plasma Count
		−0.123; *P* = 0.021	Renal Complications
	Hyperdiploidy	−0.131; *P* = 0.033	Extramedullary Disease: Count
		−0.120; *P* = 0.047	Anemia
**Disease Characteristics**	M Protein Level	−0.122; *P* < 0.001	Extramedullary Mass
		−0.124; *P* < 0.001	Extramedullary Disease: Count
		0.215; *P* < 0.001	Bone Marrow Plasma Count
		−0.478; *P* < 0.001	Albumin Level
		0.197; *P* < 0.001	β_2 Microglobulin Level
		−0.308; *P* < 0.001	Anemia
		0.084; *P* = 0.005	Neutropenia
		0.095; *P* = 0.001	Fatigue
		0.064; *P* = 0.033	Infections
		−0.080; *P* = 0.006	Bone Fractures
	Extramedullary Mass	0.845; *P* < 0.001	Extramedullary Disease: Count
		−0.079; *P* = 0.047	Bone Marrow Plasma Count
		0.151; *P* < 0.001	Serum LDH Level
		0.187; *P* < 0.001	Pain
	Extramedullary Disease: Count	−0.095; *P* = 0.018	Bone Marrow Plasma Count
		0.171; *P* < 0.001	Serum LDH Level
		0.151; *P* < 0.001	Pain
	Kappa/lambda FLC Ratio	0.075; *P* = 0.033	Bone Lesions
	Bone Marrow Plasma Count	0.163; *P* < 0.001	Serum LDH Level
		−0.159; *P* < 0.001	Albumin Level
		0.325; *P* < 0.001	β_2 Microglobulin Level
		−0.358; *P* < 0.001	Anemia
		0.118; *P* = 0.003	Renal Complications
		0.092; *P* = 0.023	Neutropenia
		0.184; *P* < 0.001	Fatigue
		0.099; *P* = 0.014	Infections
	Serum LDH Level	0.149; *P* < 0.001	β_2 Microglobulin Level
		−0.128; *P* < 0.001	Anemia
		0.116; *P* < 0.001	Renal Complications
		0.117; *P* < 0.001	Pain
		0.086; *P* = 0.006	Fatigue

	Albumin Level	−0.335; $P < 0.001$	β_2 Microglobulin Level
		0.203; $P < 0.001$	Hypercalcemia
		0.437; $P < 0.001$	Anemia
		−0.136; $P < 0.001$	Renal Complications
		−0.127; $P < 0.001$	Fatigue
	β_2 Microglobulin Level	−0.513; $P < 0.001$	Anemia
		0.725; $P < 0.001$	Renal Complications
		0.082; $P = 0.013$	Neutropenia
		0.264; $P < 0.001$	Fatigue
**Complications**	Hypercalcemia	0.087; $P = 0.003$	Anemia
		0.098; $P = 0.001$	Renal Complications
		0.075; $P = 0.031$	Bone Lesions
		0.087; $P = 0.003$	Pain
		0.061; $P = 0.036$	Bone Fractures
	Anemia	−0.278; $P < 0.001$	Renal Complications
		−0.209; $P < 0.001$	Neutropenia
		0.059; $P = 0.039$	Pain
		−0.346; $P < 0.001$	Fatigue
	Renal Complications	−0.102; $P < 0.001$	Pain
		0.146; $P < 0.001$	Fatigue
	Nervous System	−0.090; $P = 0.010$	Bone Lesions
		−0.080; $P = 0.006$	Pain
		0.087; $P = 0.003$	Fatigue
		−0.057; $P = 0.050$	Bone Fractures
	Bone Lesions	0.704; $P < 0.001$	Pain
	Neutropenia	0.082; $P = 0.005$	Fatigue
		0.134; $P < 0.001$	Infections
	Pain	0.164; $P < 0.001$	Fatigue
	Fatigue	0.076; $P = 0.010$	Infections
		0.057; $P = 0.032$	Bone Fractures
	Bone Fractures	−0.059; $P = 0.047$	Infections

Note: ECOG = European Cooperative Oncology Group; FLC = free light chain; LDH = lactate dehydrogenase; MM = multiple myeloma.

Only correlations that reached statistical significance are presented in the table. Each correlation is presented once only to avoid repetition. Proxies were used for some attributes Anemia: hemoglobin; hypercalcemia: calcium; renal complications: creatinine; nervous system; grade of neuropathy; pain: presence of at least two osteolytic lesions or a bone-related extramedullary mass; numbness and tingling: neuropathy; fatigue and infection: toxicity. Pearson's R correlation coefficients were calculated and statistical significance was set at $P < 0.05$. Data not available for the following attributes: renal comorbidities, hypodiploidy, karyotypic abnormalities, plasma cell labeling index, leukopenia, and hyperviscosity.

Appendix 2

First Round Interview Guide Topics

Part I. Disease Progression

1. How would you define disease progression over time in a treatment-free scenario?
2. Which measure(s) do you think is/are most important for disease progression of multiple myeloma?

Part II. Outcome Measures

1. Based on the literature review, we identified the following key outcome measures that are important for multiple myeloma patients: overall survival, quality of life and activities (ambulation and mobility; family and family life; friendship and social life; independence; leisure and hobbies; self-care; usual activities; sex and intimacy; work life).

   1.1. Among the identified outcomes, do you consider some of them unimportant?
   - No, all of them are important
   - Yes, some of them are not important Please specify:

   1.2. Are there other outcomes that you consider as important ones but are missing in the list?
   - No, the list is complete
   - Yes, there are some missing ones Please specify:

Part III. Attributes of Multiple Myeloma

1. Based on the literature review we identified a list of attributes (please see table below).

   1.1. For each of the variables, please comment on their importance for disease progression, symptoms and complications, and disease outcomes.

   1.2. For each variable in the following table, please suggest if they are relevant to be included in the model as important factors impacting disease progression and outcomes.

   1.3. Did we miss any relevant attributes?
   - No
   - Yes
2. Please indicate how we can split the variables into groups (i.e. symptoms/complications, patient/disease characteristics, and generic factors). For the missing attributes please specify and mention the group that they belong to.

	Group	Important for Symptoms/ Complications (yes/no)	Inclusion/ Exclusion	Important for Disease Progression (yes/no)	Important for Disease Outcomes (yes/no)	Constant Variables	Variables Changing with Disease Progression
**Anemia**						–	–
**Appetite changes**						–	–
**Bone Pain/Fracture**						–	–
**Bleeding**						–	–
**Cognitive Impairment**						–	–
**Constipation**						–	–
**Diarrhea**						–	–
**Dizziness**						–	–
**Incontinence**						–	–
**Infections**						–	–
**Kidney Damage**						–	–
**Nausea and Vomiting**						–	–
**Neuropathy**						–	–
**Pain**						–	–
**Thrombolytic Events (e.g. Stroke)**						–	–
**Weight Change**						–	–
**Age**							
**Gender**							
**Race (e.g. Hispanic, African, Black)**							
**BMI (Disease-Related Weight Loss)**							

	Group	Important for Symptoms/ Complications (yes/no)	Inclusion/ Exclusion	Important for Disease Progression (yes/no)	Important for Disease Outcomes (yes/no)	Constant Variables	Variables Changing with Disease Progression
**Comorbidities (e.g. Kidney Function, Fibrosis)**							
**Creatinine Level**							
**Albumin Level**							
**Hemoglobin Level**							
**Platelet Count**							
**ECOG Performance Status**							
**Calcium Level**							
**Testosterone Level**							
**Hypodiploidy**							
**Red Blood Cell Sedimentation Rate**							
**Serum Lactate Dehydrogenase Level**							
**Cholesterol Level**							
**Socioeconomic Status**							
**MIP-1$^{\alpha}$**							
**Subclinical Sensory Deficits**							
**Other: Please**							

	Group	Important for Symptoms/ Complications (yes/no)	Inclusion/ Exclusion	Important for Disease Progression (yes/no)	Important for Disease Outcomes (yes/no)	Constant Variables	Variables Changing with Disease Progression
**Specify______**							
**___________**							
**___________**							
**Serum IL-16**							
**Serum IL-6**							
**Marrow Microvessel Density**							
**Pro-angiogenic Cytokines (e.g. VEGF, HGF, bFGF, ANG)**							
**Subtypes (G, A, D, Kappa/ Lambda or Light Chain)**							
**sFLCR**							
**sFAS-L**							
**Serum BLyS**							
**BAFF Serum Level**							
**MTV Level**							
**Chymotrypsin Activity Levels**							
**Bone Marrow Plasma Cell Level**							
**Karyotypic Abnormalities**							

	Group	Important for Symptoms/ Complications (yes/no)	Inclusion/ Exclusion	Important for Disease Progression (yes/no)	Important for Disease Outcomes (yes/no)	Constant Variables	Variables Changing with Disease Progression
Treg Cell Count							
ISS Stage I							
ISS Stage II							
ISS Stage III							
Mayo Clinic Risk Factors							
Extramedullary Diseases							
Other: Please Specify______							
__________							
__________							
t(4;14)							
t(14;16)							
t(11;14)							
add(14q32)							
del(1p12)							
del(13p14)							
del(17p)							
c-MYC							
CD81							
IRF4							
MKI67							
GAGE Family							
MAGEC1/CT7							
del(p53)							
Ku86 & Artemis (NHEJ)							
IL-6							
CCND1							

	Group	Important for Symptoms/ Complications (yes/no)	Inclusion/ Exclusion	Important for Disease Progression (yes/no)	Important for Disease Outcomes (yes/no)	Constant Variables	Variables Changing with Disease Progression
**MDR1**							
**1p Abnormalities**							
**1q Abnormalities**							
**del(13/13q)**							
**Other: Please Specify_____**							
___________							
___________							

Notes: ANF = angiopoietin; BAFF = B-cell activating factor; bFGF = basic fibroblast growth factor; BLyS = B lymphocyte stimulator; BMI = body mass index; CCND1 = cyclin D1; CD81 = cluster of differentiation 81; ECOG = European Cooperative Oncology Group; GAGE = G antigen 1; HGF = hepatocyte growth factor; Ig = immunoglobulin; IL = interleukin; IRF4 = interferon regulatory factor 4; ISS = International Staging System; MAGE = melanoma antigen family 1A; MDR1 = multi-drug resistance gene; MIP-1α = macrophage inflammatory protein-alpha; MTV = metabolic tumor volume; MKI67 = marker of proliferation Ki-67; NHEJ = non-homologous end-joining; sFAS-L = serum FAS ligand; FLCR = serum free light chain ratio; Treg = regulatory T cell; VEGF = vascular endothelial growth factor.

3. Do you think it makes sense to separate symptoms and complications?
    - Yes
    - No
4. If we decide to separate symptoms and complications, how would you recommend to group them (please go to Table)?
5. Some of the patient and disease characteristics are constant over time, however, some characteristics change over time depending on how the disease progresses and treatment. Do you think for this reason they need to be grouped differently? Can you indicate which variables are not influenced by the progression of the disease and/or treatment, and which variables change over time?
6. How would you comment on "socioeconomic status" as one of the patient characteristics?
    6.1. Do you consider socioeconomic status an important variable to be included in the model?
    - Yes
    - No

    6.2. If you consider it as important, why is it important?
7. According to your opinion, are there attributes which are measuring the same aspect of the disease? (i.e. any duplications of attributes?) In other words, what can be the most meaningful composite measure of disease classification?
8. Do you consider it reasonable to delete some variables that can be covered by the composite measure of disease classification?
    - No, no variable should be deleted
    - Yes, some of them can be deleted Please specify:

Part IV. The Role of M Protein

9. M protein is produced in excess by an abnormal clonal proliferation of plasma cells. Please explain the role of M protein in multiple myeloma.
    9.1. Does M protein impact directly only on renal function?
    - Yes
    - No Please specify the reason:

    9.2. Do you agree with the following statement: "High M protein implies high number of cancer cell activity which causes non-renal complications and symptoms"?
    - Yes
    - No Please explain:

    9.3. Is M protein alone the best way to measure how multiple myeloma progresses over time?
    - Yes
    - No Please explain:

    If M protein is not the measure for disease progression by itself, to which group of variables does it belong?

Closure

1. Relook at the conceptual model. Do you think that there are any missing attributes or parameters?
